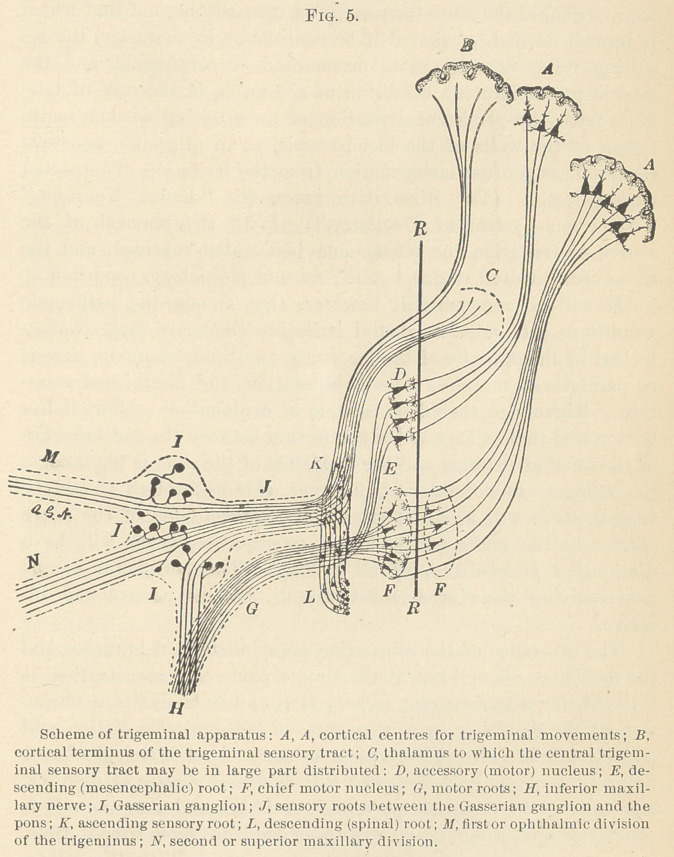# The Neuron Conception as a Means of Interpreting Reflex Disorders Due to Dental Irritation

**Published:** 1899-08

**Authors:** Albert P. Brubaker

**Affiliations:** Philadelphia


### International Dental Journal.

VOL. XX. AUGUST, 1899. NO. 8.

### Original Communications.[1]

### THE NEURON CONCEPTION AS A MEANS OF INTERPRETING REFLEX DISORDERS DUE TO DENTAL IRRITATION.[2]

BY DR. ALBERT P. BRUBAKER, PHILADELPHIA.

SEVERAL years ago I collated and published ("American System of Dentistry," vol. iii. p. 435) a series of clinical cases, medical, dental, and neurological, which it was believed for many reasons were reflex in origin, the result of abnormal irritation present in the teeth or in their associated structures. In this paper no attempt was made to classify these cases on the basis of their pathology. They were arranged merely for convenience of presentation and consideration into three groups as follows:

1. Affections of peripheral organs,—*e.g.*, ocular, aural, muscular, visceral, and vasomotor.

2. Affections of nerves and subordinate nerve-centres,—*e.g.*, facial and other neuralgias, paresis, paralyses, and tetanus.

3. Affections of cerebral centres,—*e.g.*, headache, epilepsy, chorea, hysteria, and insanity.

---

[1] The editor and publishers are not responsible for the views of authors of papers published in this department, nor for any claim to novelty, or otherwise, that may be made by them. No papers will be received for this department that have appeared in any other journal published in the country.

[2] Read before the Academy of Stomatology, February, 1899.

A study, however, of these cases in the light of modern knowledge makes it apparent that they may be classified in a manner more in accordance with physiologic processes. The visible phenomena may be either *motor,* relating to muscles, *vasomotor,* relating to the vascular apparatus, or *secretory,* relating to glands. Again, any motor, vasomotor, or secretory phenomenon may be either one of increased activity, as muscular spasm, contraction of blood-vessels, increased secretion, or one of decreased activity, as muscular paralysis, dilatation of blood-vessels, and diminished secretion. The invisible phenomena are sensory in character. The sensations experienced by the individual possess varying degrees of intensity, varying periods of duration, followed by periods of suspension, even when the cause which has been operative has not been removed and is apparently in continuous action.

It was also stated in the paper alluded to that no attempt was made to explain the mechanism of a normal reflex action, or the derangements of mechanism by which these pathologic conditions were brought about. This for the reason that the histologic and physiologic facts at command were not sufficient to enable me to present a satisfactory theory, at least as to the nature of either the normal or abnormal process.

Within recent years a number of very interesting and instructive facts relating to the histology of the nervous system have been disclosed, which, if we are correct in our interpretation of them, throw much light on the nature of reflex action, both under normal and abnormal conditions.

In the paper which I present this evening, entitled "The Neuron Conception as a Means of interpreting Reflex Disorders due to Dental Irritation," an attempt will be made to briefly place these facts before you, and then apply them to the interpretation of the pathologic conditions under consideration. In order that the problem may be made somewhat clearer, a brief reference to what is comprehended by the term reflex action may not be inappropriate. All the muscular movements of the body are caused by the transmission to the muscles of nerve impulses or nerve energy, the path of transmission being the anterior or efferent roots of the spinal nerves and the efferent roots of the cranial nerves. The source of the energy in all instances is the nerve-cells residing in the gray matter of the spinal cord and medulla oblongata, and with which all the efferent fibres are connected. The discharge of energy by these cells and its transmission to the muscles is invariably followed by a contraction of greater or less intensity, in accordance with the amount of the energy the muscles receive.

The nerve-cells, however, do not in themselves possess spontaneity of action, but require for their activity a stimulus, which may come either from the brain or the general periphery of the body. If the stimulus comes from the brain, the resultant contraction is termed a direct or voluntary action. If it comes from the periphery, it is termed an indirect, involuntary, or reflex action. (Fig. 1.) Though these two forms of activity interblend in the general activities of the body, the former may be eliminated by the induction of sleep, by narcosis from chloroform, ether, morphine, etc., or, in the case of the lower animals, like the frog, by the removal, under anæsthesia, of the brain itself. A frog so prepared will be incapable of executing spontaneous movements, and will remain perfectly passive for an indefinite period of time. When a frog, under such circumstances, is suspended and an irritation—*e.g.,* a weak solution of sulphuric acid—be applied to the skin of the toes, there will follow in a few seconds a contraction of the muscles of the leg, which will continue for a varying period of time, as long as the irritant is active. With the prompt removal of the acid the muscular contraction as speedily subsides. Increasing strengths of the acid solution induce correspondingly vigorous contractions. (An experiment illustrating these statements was then made.) The explanation of such a phenomenon may be given, in a general way, as follows: The acid, in consequence of the chemical changes it induces in the skin and the associated nerve-endings, gives rise to nerve impulses, which are transmitted through the posterior or afferent roots of the spinal nerves to the spinal cord and received by the nerve-cells; these latter, in consequence of the stimulation, discharge nerve impulses, which are transmitted to the muscles, which in turn contract. The muscular action, being the result of a stimulation reflected from the periphery (skin), is termed a reflex action. Coincident with the muscular contraction, in various regions of the body there is frequently associated a variation in the caliber of the blood-vessels and an output of the secretion of different glands. The stimulation of the sensory nerves of the mucous membrane of the stomach by food induces a reflex contraction of the muscular walls of the stomach, a dilatation of the blood-vessels, and an output of gastric juice from the gastric glands. Exactly similar reflex actions may and do take place constantly through the sensory and motor cranial nerves. The trifacial nerve, on account of its extensive peripheral distribution as well as its central connections with all the cranial motor nerves, is intimately related to and connected with a very large number of normal as well as abnormal reflex actions.

The structures involved in all reflexes may be summarized as follows:

1. Sentient surface,—skin, mucous membrane, and sense organ.
2. An afferent nerve.
3. An emissive cell, from which arises—
4. An efferent nerve, distributed to a responsive organ, as
5. Muscle, gland, blood-vessel, etc. (Fig. 2.)

As simple, apparently, as the mechanism of a normal reflex action is, its interpretation was a matter of much difficulty not a

FIG. 2.

Diagram illustrating reflex action: *S*, sentient surface from which proceeds the afferent nerve; *M C*, motor or emissive cell giving origin to an efferent nerve, which terminates in *M; M*, motor organ; *G*, ganglion on posterior root.

very long time ago. Still more difficult was it to interpret its inhibition or its irregular manifestations. At the present time, with new facts relating to the structure of nerve-cells, the problems are somewhat clearer.

THE NEURON.

Within quite recent times the old conception that the nervous system consisted of two distinct histologic elements,—nerve-cells and nerve-fibres,—which not only differed in their mode of origin, their properties, their relation to each other, and their functions, has been entirely disproven. The nerve-tissue has been resolved by the investigations of modern histologists to a single morphologic unit, to which the term neuron has been given. The entire nervous system has been shown to be but an aggregation of an infinite number of neurons, each of which is histologically distinct and independent. The neuron or neurologic unit is histologically a nerve-cell, the surface of which presents a greater or less number of processes in varying degrees of differentiation. As represented in Fig. 3, the neuron may be said to consist of: 1, the nerve-cell, neurocyte or corpus; 2, the nerve-process or axon; 3, the end tufts or terminal branches.

Fig. 3.

A, efferent neuron; B, afferent neuron.

The nerve-cell, or the body of the neuron, presents a variety of shapes and sizes in various parts of the nervous system. From the surface of the adult cell portions of its protoplasm are projected in various directions, which, rapidly dividing and subdividing, form a series of branches termed *dendrites* or *dendrons.* In some situations the ultimate branches of the dendrites present short lateral processes or buds, known as *lateral buds* or *gemmules,* which impart to the branches a feathery appearance. This characteristic is common to the cells of the cortex of the cerebrum and cerebellum. The ultimate branches of the dendrites, though forming an intricate felt-work, never anastomose with one another nor unite with dendrites of adjoining cells. According to the number of axons nerve-cells are classified as monaxonic, diaxonic, or polyaxonic. Most of the cells of the higher vertebrates are monaxonic. In the ganglion of the posterior roots of the spinal and cranial nerves, however, they are diaxonic. In this situation the axons, emerging from opposite poles of the cell, either remain separate and pursue opposite directions or unite to form a common stem, which subsequently divides into two branches, which then pursue opposite directions.

The *axon* or nerve-process arises from a cone-shaped projection from the surface of the cell, and is the first outgrowth from its protoplasm. At a short distance from its origin it becomes markedly differentiated from the dendrites, which subsequently develop. It is characterized by a sharp, regular outline, a uniform diameter, and a hyaline appearance. At a variable distance from the cells the axons, especially those forming the spinal nerve system, become invested with a myelin sheath and a neurilemma, thus constituting what is known as a medullated nerve-fibre. The end tufts, or terminal branches, are formed by the splitting of the axon into a number of filaments, which remain independent of one another, and are free from medullary investment. The histologic peculiarities of the terminal organs vary in different situations, and in many instances are quite complex and characteristic. In peripheral organs, as muscles, glands, blood-vessels, skin, mucous membrane, etc., the tufts are in direct organic connection with their cellular elements. In the central nervous system the tufts are in more or less intimate relation with the dendrites and adjacent neurocytes.

From this point of view, then, all the cranial motor nerves and the anterior roots of the spinal nerves are neurons, whose bodies or neurocytes with their system of dendrites are situated in the gray matter of the medulla oblongata and spinal cord, and from which axons have grown outward to put themselves into relation with peripheral organs, as muscles, blood-vessels, glands, etc. Having but one axon which transmits nerve force outward, they are termed monaxonic efferent neurons.

In like manner all the cranial sensory nerves and the posterior roots of the spinal nerves are neurons, whose bodies or neurocytes are found in the ganglia of the posterior roots, and from which two axons primarily develop, one of which grows outward towards the periphery (skin and mucous membrane), the other grows inward to put itself, by means of its terminal branches, into intimate physiologic relation, at least, with the dendrites of the adjoining neurocytes. Having two axons which transmit nerve force inward, they are known as diaxonic afferent neurons. A study of the developmental stages of these afferent or sensory nerves (Fig. 4) shows that they develop from the ganglia, practically outside of the central nervous system, and only subsequently become connected with it. This fact has an important bearing on the relations of the fifth nerve to its associated structures.

FIG. 4.

Diagram showing the mode of origin of the anterior and posterior roots, or the efferent and afferent neurons.

THE FIFTH OR TRIFACIAL NERVE AS A COLLECTION OF NEURONS.

The fibres which collectively make up the fifth or trifacial nerve must be considered, in the light of present knowledge, as having their origin in the Gasserian ganglion rather than in the medulla oblongata. (Fig. 5.) This ganglion is to be regarded as an enormous collection of neurocytes embedded in connective tissue, each of which gives origin, in the early stages of development, to two axons, which, however, soon shift their position and blend together to form a common stem. This stem, at a short distance from the neurocyte, then divides into two portions, one of which passes towards the periphery to enter into the formation of the ophthalmic, the superior or inferior maxillary division, as the case may be; the other passes towards the medulla oblongata, where its terminal branches come into physiologic relation with the dendrites of the neurocytes or nerve-cells, which give origin to the axons or nerve-fibres, which constitute the various cranial motor nerves or efferent neurons. It is quite probable that subsequent investigations will demonstrate the truth of the supposition that the different peripheral sensory areas, to which the different branches of the fifth nerve are distributed, will be found to be in direct physiologic connection with muscular, vascular, and secretory areas through related efferent fibres. With this anatomical arrangement in mind, it becomes possible to interpret normal as well as abnormal reflexes.

A stimulus, for example, whether mechanical, chemical, or physical, applied to any one of the peripheral terminal branches, will, as is well known, develop a series of nerve impulses, which will be transmitted through corresponding nerve-fibres to the medulla, where they will be distributed by the central terminal branches. These nerve impulses will be then taken up by the dendrites of the related neurocytes or nerve-cells, by which in turn they are transmitted to the peripheral organs, with the production of a movement. Should the stimulus possess an intensity beyond that which is termed normal, or should it be continuous in character, the resulting reflex becomes more pronounced or continuous, and the muscle passes into the condition of spasm, as in the case of trismus, from the persistent irritation of an erupting wisdom-tooth, spasm of the walls of the blood-vessels, as in migraine, excessive secretion, as profuse lachrymation from the irritation of impacted cuspid teeth. (For illustrative cases, see "Reflex Neuroses," "American System of Dentistry.") With the removal of the source of irritation the phenomena just stated subsided, and the structures involved return to their normal physiologic condition.

It will be remembered, however, that among the pathologic conditions attributed to dental irritation there are some characterized by the opposite of the foregoing condition,—namely, paresis or paralysis of muscles, vascular dilatation, and diminished secretion. These also should be capable of explanation. Now, it has been stated that as long as the connection between the end branches of the afferent neurons and the dendrites of the cells is physiologic the reflexes are normal. If, however, this connection should be broken, there will be no means for the transmission of the nerve energy to the nerve-cells, and, in consequence, there will be a diminution or abolition of all reflexes. The peripheral organs not receiving their accustomed stimuli, become passive and inactive.

The severance of the connection between the end branches and the dendrites, though not at the time capable of demonstration, is believed, for many reasons, to be a fact, and to be due to a retraction of the dendrites in consequence of excessive stimulation and activity of the cell. The neuron is not a fixed histologic but a living physiologic unit, endowed with a certain degree of motility, especially in its dendritic portions. It is believed that the cell is capable of retracting and extending its dendrites from time to time, in accordance with its physiologic necessities. With the retraction of the dendrites nerve impulses cannot be transmitted or received, and physiologic action must of necessity cease. That it is the dendrites that are retracted rather than the terminals of the afferent neurons is rendered probable by the fact that the individual cannot execute a voluntary movement with the muscles which are in the condition of paresis or paralysis, which would otherwise be the case.

In the early part of this paper it was stated that among the results of abnormal dental irritation are sensations of pain, possessing varying degrees of intensity, varying periods of duration, followed by periods of cessation, even when the pathologic condition of the teeth remains the same, and in apparently continuous action. An explanation of these facts is afforded by reference to the same mechanism. When nerve impulses, arising in consequence of peripheral irritation, are transmitted by the afferent nerves to the central nervous system, they are received by the dendrites of nerve-cells, and transmitted to the cerebral cortex. With the reception of the nerve impulses by these cells and their dendrites, a molecular disturbance at once takes place. Coincident with this disturbance there arises that which is termed a sensation, the intensity of which will depend on the strength of the primary stimulus. Though the sensation is a *psychical* fact, the mind refers the sensation, as a rule, to the point of stimulation, though this is not invariably the case. It is a well-known fact, for example, that the mind refers the sensation of toothache not infrequently to the side of the jaw opposite to that of the lesion, as well as to other regions of the face and head. If the stimulus at the terminal of the trifacial nerve is continuous, and if it possesses sufficient intensity, the resulting sensation acquires that degree of intensity characteristic of trifacial neuralgia. With the removal of the irritation the cell stimulation and activity subside and the painful sensation disappears. Should the nerve-cells of the cerebral cortex become exhausted and retract their dendritic processes, their functional activity, characterized by the production of a sensation, would cease even though the peripheral irritation remained unchanged. With the restitution of the nerve-cells and an extension again of their dendrites, there would be a re-establishment of the physiologic connection, after which the cells would resume their original activity, with a return of the painful sensations.

Though the conception of the neuron may in the future be subject to some modifications, yet it can hardly be doubted that the histologic or physiologic facts which have been discovered are in the main true. With the progress of investigation into the mode of activity of neurons in general many hitherto obscure and complex phenomena, both normal and pathologic, will gradually be interpreted and made clear. It was with this idea that the foregoing pages were presented this evening. The conception of the trifacial nerve as a collection of individual afferent neurons, the origin of which is in the Gasserian ganglion, and the central terminal branches of which are in physiologic connection both with efferent motor centres and with cerebral sensory centres,—this anatomic conception, taken in connection with what is known as to the physiologic action of neurons in general, will enable any one to more accurately interpret all of the ordinary reflexes which fall under the notice of the dental practitioner.

## Figures and Tables

**Fig. 1. f1:**
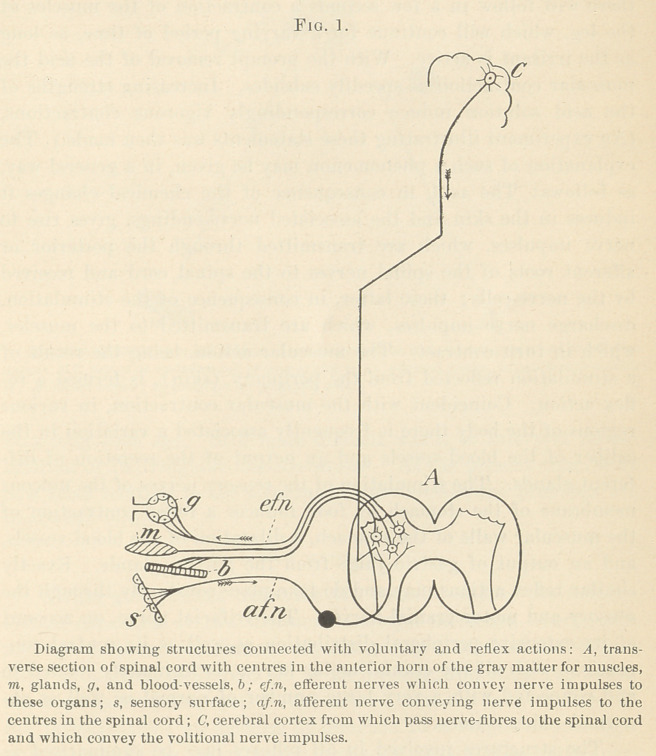


**Fig. 2. f2:**
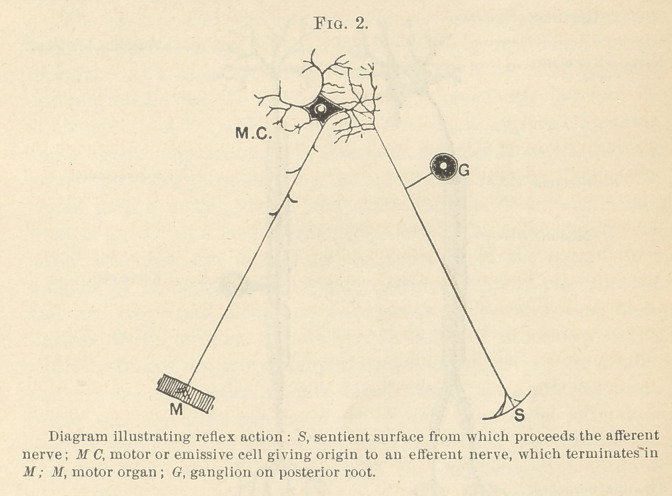


**Fig. 3. f3:**
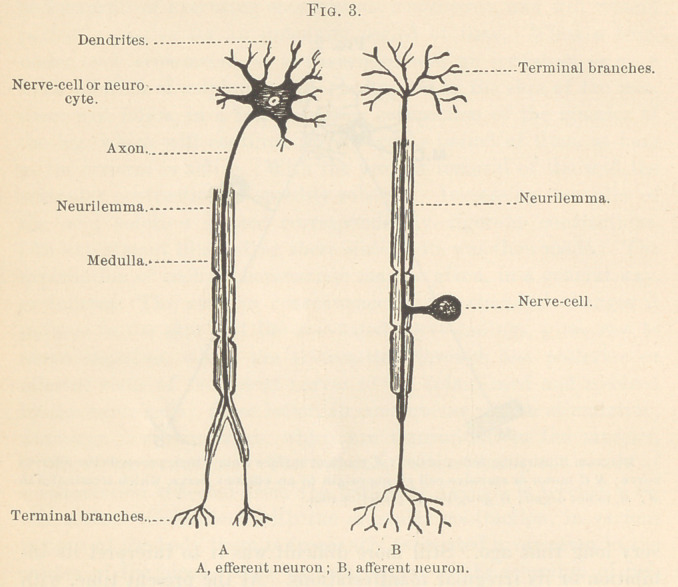


**Fig. 4. f4:**
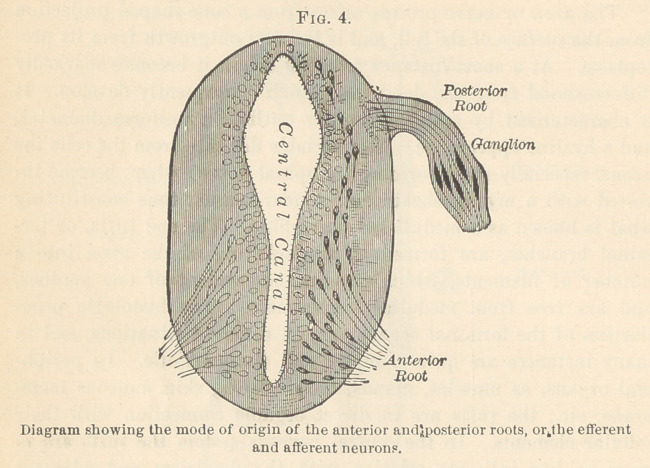


**Fig. 5. f5:**